# Letter to the Editor: Intellectual conflicts of interest in spinal cord stimulation research - The gap between theoretical knowledge and clinical expertise

**DOI:** 10.1016/j.inpm.2025.100715

**Published:** 2025-11-30

**Authors:** Hemant Kalia, Scott Pritzlaff, Konstantin Slavin

**Affiliations:** a Center for Research & Innovation in Spine & Pain, Rochester, NY, USA; b Department of Anesthesia & Pain Medicine, UC Davis Health, Sacramento, CA, USA; c Department of Neurosurgery, University of Chicago, Chicago, IL, USA; d International Neuromodulation Society, USA

To the Editor,

We read with interest the recent systematic review by Przybysz et al. titled “A Systematic Review of Evidence Comparing Spinal Cord Stimulation with Conventional Medical Management for Persistent Spinal Pain Syndrome - Type 2.” [1] We commend the authors for their careful effort to synthesize the available randomized controlled trial (RCT) evidence for this important clinical issue. The authors deserve recognition for following established best practices in evidence synthesis. The clear description of PICOS (Population, Intervention, Comparison, Outcomes, and Studies) criteria, the rigorous application of the Grades of Recommendation, Assessment, Development, and Evaluation (GRADE) framework, and the use of Cochrane Risk of Bias tools demonstrate a commitment to methodological transparency and quality assessment.

However, despite this procedural rigor, the review exhibits a profound disconnect between the evidence presented and the conclusion drawn, especially regarding the comparison between burst spinal cord stimulation (SCS) and sham stimulation. Their findings raise significant concerns about intellectual conflicts of interest (COI), a situation in which investigators with strong theoretical and methodological expertise may at times overlook the nuances that come from direct clinical or practical experience in the field [2,3]. Intellectual COIs are frequently underreported compared to financial COIs, yet they can substantially influence the framing of research questions, study design, and interpretation of findings [[4], [5], [6]]. For example, systematic review teams composed primarily of methodologists without adequate clinical expertise may inadvertently misinterpret clinical nuances, leading to conclusions that do not fully reflect real-world practice. Conversely, excessive reliance on clinical experts without methodological independence can also introduce bias [7].

## 1 Defining intellectual conflict of interest and its manifestation as confirmation bias

Traditional COI frameworks focus primarily on financial relationships between researchers and industry sponsors. However, intellectual COIs represent an equally problematic but often overlooked bias that emerges from the gap between theoretical knowledge and practical clinical expertise.

This intellectual loyalty often shows through well-described cognitive biases. The most important of these is confirmation bias, which is the tendency to focus on information that supports one's existing beliefs while questioning or dismissing evidence that contradicts them. A related bias, known as anchoring bias, happens when people give too much weight to the first piece of information they come across [8]. In this case, it is the long history of positive but often methodologically weaker studies on SCS.

## 2 The real-world impact: how payors weaponize flawed systematic reviews

Insurance companies and health technology assessment groups, such as the Institute for Clinical and Economic Review (ICER) in the United States and the National Institute for Health and Care Excellence (NICE) in the United Kingdom, often use systematic reviews as key evidence when making coverage decisions. When these reviews contain major clinical misunderstandings, such as including flawed studies like the Hara trial, they can be misused by payors to justify restricting access to costly but effective treatments [9,10].

The healthcare landscape already faces significant barriers to pain care, as prior authorization requirements for non-opioid treatments continue to cause delays and add to administrative burdens. Denials of coverage have increased sharply, with some insurers rejecting a large share of claims, yet only a small number of patients pursue appeals. Even more concerning, most appealed denials are ultimately overturned, revealing the arbitrary nature of many initial decisions [11].

## 3 The clinical context: lessons from the hara study exclusion

The authors' systematic review notably omitted several key publications that directly challenged the methodology and conclusions of the Hara study. These omissions are especially concerning because leading neuromodulation experts who have extensively critiqued the Hara study for fundamental methodological flaws have raised these issues. The critiques, published in reputable journals such as *Neuromodulation,* [[12], [13], [14], [15]] *Pain Practice* [16,17], and through letters to *JAMA* [[18], [19], [20], [21], [22]], highlighted numerous concerns, including but not limited to:

1. The trial used a nonstandard 4-spike burst waveform at low sub-paresthesia amplitudes, which prior evidence shows is equivalent to sham and not representative of real-world clinical practice or manufacturer recommendations.
2. Patient trialing was performed with tonic stimulation, yet permanent implants received burst therapy, introducing selection bias and mismatching trial and treatment arms.
3. The trial's analgesic criteria were lax (2-point or 30 % reduction), and outcome measures did not align with those typically used for spinal cord stimulation, compromising responder classification.
4. Devices were not optimized for individual patients, and the use of blinded, fixed stimulation settings further prevented therapy adjustments, diverging from routine care.
5. The study's design, conduct, and reporting (including unclear blinding, lead positioning, and insufficient washout) were widely criticized, rendering its conclusions neither generalizable to contemporary SCS protocols nor valid for clinical or payor policy.

## 4 The intellectual conflict of interest in evidence synthesis

The exclusion of these expert critiques from a systematic review represents a form of intellectual bias that occurs when methodologists ignore the granular details in spinal cord stimulation and fail to appreciate the clinical significance of study limitations. This creates several problems:

*Methodological Rigor Without Clinical Context:* Although the authors demonstrate excellent systematic review methodology, their apparent lack of familiarity with nuanced neuromodulation clinical practice led them to include studies with fundamental design flaws and omit important critical analyses by clinical experts.

*Perpetuation of Flawed Evidence:* By including the Hara study without acknowledging its extensive critiques, the authors inadvertently legitimize research that the clinical neuromodulation community has largely rejected due to its methodological shortcomings.

*Impact on Clinical Practice:* Systematic reviews carry significant weight in evidence-based medicine and influence payor policies. When conducted without adequate clinical input, they can inappropriately restrict patient access to effective treatments.

## 5 Conclusion

Methodological rigor alone is not enough. When systematic reviews of complex therapies are done without clinical insight, they may produce conclusions that are statistically valid but clinically irrelevant. In neuromodulation, credible evidence requires both methodological accuracy and practical understanding of how treatments work in real-world settings. This includes understanding programming, waveforms, and the subtle nuances of these complex therapies.

Authors of systematic reviews carry significant responsibility. Their conclusions influence coverage decisions and ultimately determine whether patients have access to care. Including flawed studies without adequate clinical interpretation allows insurers and health technology assessment organizations to misuse those findings to justify denials. The harm falls on patients who lack the resources to appeal or pay for treatment on their own.

The critiques of the Hara trial have identified serious methodological and ethical flaws, such as protocol deviations, the use of nonstandard stimulation settings, and inadequate blinding. Ironically, *Interventional Pain Medicine* published this systematic review that heavily relied on such a flawed study, even though its affiliated society, the International Pain and Spine Intervention Society (IPSIS), had already issued a detailed research division critique highlighting these issues [23].

Systematic reviewers must bring clinical judgment back into evidence-based medicine. The goal is not to satisfy methodological ideals but to produce evidence that accurately reflects patient care and supports sound clinical decision-making.

## Declaration of competing interest

The authors declare the following financial interests/personal relationships which may be considered as potential competing interests:Hemant Kalia MD MPH reports a relationship with Abbott that includes: consulting or advisory and travel reimbursement. Hemant Kalia MD MPH reports a relationship with Nalu Medical Inc that includes: consulting or advisory. Hemant Kalia MD MPH reports a relationship with Curonix LLC that includes: consulting or advisory. Hemant Kalia MD MPH reports a relationship with nervonik that includes: consulting or advisory. Hemant Kalia MD MPH reports a relationship with SPR Therapeutics Inc that includes: consulting or advisory. If there are other authors, they declare that they have no known competing financial interests or personal relationships that could have appeared to influence the work reported in this paper.
